# Colorectal Cancer Risk With Negative Colonoscopy or Nonadherence After Positive FOBT Screening

**DOI:** 10.1001/jamanetworkopen.2026.2404

**Published:** 2026-03-19

**Authors:** Hanna Heyman, Deborah Saraste, Håkan Jonsson, Johannes Blom

**Affiliations:** 1 Department of Surgery, Södersjukhuset, Stockholm, Sweden; 2 Department of Clinical Science and Education, Karolinska Institutet, Stockholm, Sweden; 3 Department of Epidemiology and Global Health, Umeå University, Umeå, Sweden

## Abstract

**Importance:**

Population-based colorectal cancer (CRC) screening programs are implemented globally. When determining screening intervals, variations in the risk of subsequent CRC by colonoscopy outcome should be considered.

**Objective:**

To evaluate the incidence of CRC after a negative screening colonoscopy result or nonadherence to colonoscopy in individuals with a positive fecal occult blood test (FOBT) result.

**Design, Setting, and Participants:**

This was a prospective cohort study conducted between January 1, 2008, and December 31, 2021. It was conducted within a population-based CRC screening program in the Stockholm-Gotland region, Sweden, using biennial FOBT as the primary screening test in the target population aged 60 to 69 years. The study cohort included all men and women born between 1938 and 1954 residing in the Stockholm-Gotland region between 2008 and 2012 who were invited to a CRC screening. All participants with a positive FOBT result were identified. Data provided by national registers were analyzed from October 2024 through January 2026.

**Exposures:**

Individuals with a positive FOBT result were categorized as (1) having a negative result on recommended follow-up colonoscopy or (2) nonadherence to recommended follow-up colonoscopy.

**Main Outcomes and Measures:**

The primary outcome was CRC incidence compared with the general population (defined as all individuals in the cohort who were invited to screening, excluding those with negative colonoscopy or nonadherence following positive FOBT). This was measured by standardized incidence ratio (SIR).

**Results:**

Of 318 096 individuals invited to CRC screening with FOBT, 14 873 had a positive FOBT result (7799 male [52.4%]; median [IQR] age, 65 [63-67] years). Of these individuals, 11 473 (87.3%) underwent a colonoscopy, and 8433 of those colonoscopies (73.5%) were negative. Individuals with a negative screening colonoscopy result had a significantly lower observed CRC incidence (SIR, 0.52; 95% CI, 0.39-0.68) relative to the general population, with a lower SIR in men (0.37; 95% CI, 0.25-0.56) than women (0.71; 95% CI, 0.49-1.03). In contrast, individuals with a positive FOBT who did not adhere to follow-up colonoscopy had a markedly increased CRC incidence (SIR, 4.21; 95% CI, 3.24-5.48) compared with the general population.

**Conclusions and Relevance:**

In this study, a decrease in CRC incidence was observed among individuals with negative results in screening colonoscopies, with a greater decrease in men, supporting risk-based, individualized follow-up strategies. A high-risk group for CRC was identified among individuals who did not adhere to follow-up colonoscopy, enabling targeted interventions to improve early CRC detection.

## Key Points

**Question:**

What is the risk of colorectal cancer after a negative colonoscopy result or after nonadherence to follow-up colonoscopy among individuals with a positive result in a biennial fecal occult blood test screening?

**Findings:**

In this cohort study of 14 873 individuals with a positive fecal occult blood test screening test, those with a negative screening colonoscopy result had a decreased colorectal cancer risk, while nonadherence to follow-up colonoscopy was associated with an increased risk compared with the general population.

**Meaning:**

These findings support risk-based follow-up strategies in colorectal cancer screening programs and highlight a high-risk group that may benefit from targeted interventions to improve early tumor detection.

## Teaser

This cohort study investigates the risk of colorectal cancer associated with a negative colonoscopy result or nonadherence to follow-up colonoscopy after a positive fecal occult blood test (FOBT) screening result.

## Introduction

Population-based CRC screening programs using fecal occult blood test (FOBT) are implemented globally, but reinvitation intervals after a negative follow-up colonoscopy result (ie, with no diagnosed CRC or advanced adenoma requiring surveillance) vary across countries. For instance, Sweden and England continue biennial screening invitations, while Denmark and the Netherlands suspend invitations for 8 to 10 years after a negative screening colonoscopy result.^1,2,3^ The program variability reflects conflicting evidence in current research.

Colonoscopy resources are limited, and the procedure may cause complications and harm among screening participants. Furthermore, the screening program must be cost-effective. Only a small percentage (approximately 2%) of individuals are recalled for follow-up colonoscopy after a positive FOBT result. However, costs associated with these colonoscopies exceed twice the total cost of the entire FOBT program.^4^ Therefore, it is essential to use endoscopic resources as efficiently as possible. Even though colonoscopy is effective in diagnosing CRC, some cases are still diagnosed after a negative colonoscopy result; these are referred to as postcolonoscopy colorectal cancer.^5^

Several studies have demonstrated a reduced risk of CRC extending beyond 10 years after a negative colonoscopy result.^6^ However, evidence within the context of population-based CRC screening programs remains limited. To address this gap, we conducted a register-based prospective cohort study within the Stockholm-Gotland CRC screening program in Sweden. We aimed to evaluate the risk of CRC among individuals who did or did not undergo follow-up colonoscopy after a positive screening FOBT result.

## Methods

This cohort study was approved by the Swedish Ethical Review Authority and was conducted according to the Declaration of Helsinki guidelines. Because all data were pseudonymized, the requirement for obtaining informed consent was waived. Reporting of this research follows the recommendations of the Strengthening the Reporting of Observational Studies in Epidemiology (STROBE) reporting guideline.

### Screening Program

The CRC screening program in the Stockholm-Gotland region of Sweden, targeting individuals at mean risk aged 60 to 69 years without exclusions, was initiated in 2008.^7^ Each birth cohort was assigned specific years to initiate early or late screening or was not invited to screening.^8^ Invitations were mailed biennially and included an FOBT with a prepaid return envelope. Individuals with a positive test result were referred for diagnostic follow-up colonoscopy at the endoscopy clinic within their catchment area. The screening program has been described in detail previously.^8^

Initially, guaiac-based FOBT (gFOBT; Hemoccult test; Beckman Coulter) was used. In October 2015, gFOBT was replaced with a fecal immunochemical test (FIT; OC-Sensor; Eiken Chemical Co) with the cutoff for a positive test set at 40 and 80 μg Hb/g of feces for women and men, respectively.^9^ All screening information, including invitations, participation, test results, and colonoscopy outcome data, was recorded in the Register for Colorectal Cancer Screening, maintained by the Regional Cancer Center of Stockholm-Gotland, until March 2019, when the system transitioned to the Swedish Quality Register for Colonoscopies and Colorectal Cancer Screening (SveReKKS). All endoscopists performing screening colonoscopies were required to meet minimum standards, including a cecal intubation rate of at least 90%, at least 150 colonoscopies performed annually, and an adenoma detection rate (ADR) of at least 25%.^1^

### Study Cohort

The target population included all 318 096 men and women residing in the Stockholm-Gotland region between 2008 and 2012 born between 1938 and 1954 who were invited to CRC screening. We identified the first positive test result (gFOBT or FIT) among all invited participants and categorized them into 2 cohorts: those who underwent follow-up colonoscopy during the study period and those who did not adhere to follow-up colonoscopy. A positive colonoscopy result was defined as an examination with significant findings warranting referral to a surgical unit (ie, suspected cancerous lesions or adenomas unsuitable for endoscopic removal) or a postpolypectomy surveillance program (ie, adenomas with high-grade dysplasia, the presence of >5 adenomas, or adenomas >10 mm in size).^10^ A negative colonoscopy result was defined as an examination with no CRC or other findings requiring surgery or postpolypectomy surveillance.

### Data Retrieval

From the Register for Colorectal Cancer Screening and SveReKKS, data on screening invitations, participation, and quantitative FIT values were collected. Data on adherence with follow-up colonoscopy and colonoscopy quality were also collected, including bowel preparation (assessed by the endoscopist), cecal intubation, withdrawal time (recorded when >10 minutes), and endoscopic photo documentation of the cecum. Bowel preparation, cecal intubation, and adequate withdrawal time are recognized as key performance indicators for colonoscopy.^11^

Information on incident CRC diagnoses was retrieved from the Swedish National Cancer Register of the National Board of Health and Welfare. The identified *International Classification of Diseases, Seventh Revision* (*ICD-7*) codes were 153.X (malignant neoplasm of large intestine, except rectum) or 154.0 (malignant neoplasm of rectum) excluding morphology (histology) codes C24; 091 (neuroendocrine tumor), 093 (lymphoma), 094 (adenoma), 144 (squamous cell carcinoma), or 793 (gastrointestinal stromal tumor). The coverage rate of the Swedish Cancer Register is approximately 99%.^12^ For each CRC case, data were collected on sex, date of index screening test, quality of index colonoscopy, date of CRC diagnosis, age at diagnosis, and screening participation up to the time of diagnosis. The Swedish Colorectal Cancer Registry was used to collect information on tumor stage and location.^13^ If the CRC diagnosis was registered within 6 months of the mailing date of an FOBT test, the CRC was classified as a screen-detected cancer.^5^ The unique national registration number assigned to every Swedish citizen was used to link registries.

### Statistical Analysis

Age-standardized incidence ratio (SIR) was used to compare the risk of CRC after a negative colonoscopy and among individuals who did not undergo colonoscopy after a positive FOBT with the general population as reference. Follow-up started 6 months after the mailing date of FOBT and continued until CRC diagnosis, death, emigration, or December 31, 2021, whichever occurred first. The general population was defined as all individuals in the cohort who were invited to screening, excluding the 2 groups described previously. This group included screening participants and nonparticipants and was used as the reference group to represent the general population in a region with population-based screening. For the reference group, the follow-up started 6 months after the first screening invitation (the mailing date of the FOBT). A sensitivity analysis compared incidence in the general population, restricted to screening participants, with negative colonoscopy and nonadherence subgroups. Incidence rates were calculated as events per 100 000 person-years. Cumulative hazards were estimated using the Nelson-Aalen method for participants with a negative colonoscopy and those who did not adhere to colonoscopy.

Differences in median FIT values by colonoscopy results (positive vs negative) and sex were assessed using the Wilcoxon rank-sum test. The adenoma detection rate was defined as detection of at least 1 nonadvanced adenoma among negative colonoscopies. Available-case analysis was done to handle missing data. A sensitivity analysis was performed, restricting the cohort to negative colonoscopy results in which all quality indicators were fully met. We used R statistical software version 4.2.2 (R Project for Statistical Computing) for all calculations. Statistical significance was defined as *P* < .05 for a 2-sided hypothesis. Data were analyzed from October 2024 through January 2026.

## Results

A total of 318 096 individuals were invited to CRC screening, and 230 812 individuals (72.6%) participated at least once. Among 14 873 individuals (7799 male [52.4%]; median [IQR] age, 65 [63-67] years) with a positive screening test result (6.4%), 11 473 individuals (87.3%) underwent a follow-up screening colonoscopy and 1662 individuals (12.7%) did not. A change in the registration procedure in 2019 led to a reclassification of variables, with 1738 individuals recorded under a different protocol, including a revised categorization of negative colonoscopy results and referral back to the screening program. To reduce the risk of misclassification bias, these cases were excluded from the study. Of all colonoscopies, 8433 results (73.5%) were classified as negative (Figure). A total of 1662 individuals had a positive screening test result but did not undergo the follow-up colonoscopy. Quality indicators were met in more than 90% of screening colonoscopies, with similar results observed in individuals with negative colonoscopy results and those with positive findings (Table 1). Among quality indicators, documentation of a photo of the cecum had the lowest completion rate (7739 colonoscopies [91.8%]), whereas a withdrawal time longer than 10 minutes had the highest completion rate (8354 colonoscopies [99.1%]). The adenoma detection rate for nonadvanced adenomas was 3010 of 8430 colonoscopies with negative results (35.7%). The longest possible follow-up was 13.5 years, and the total follow-up in the 2 groups was 71 151 person-years.

**Figure.**
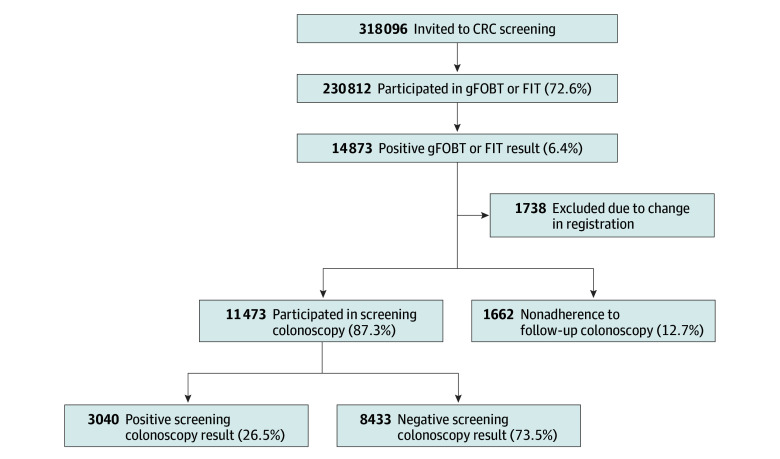
Study Flowchart The flowchart demonstrates the study cohort derived from the population-based colorectal cancer (CRC) screening program in the region of Stockholm-Gotland, Sweden. gFOBT indicates guaiac fecal occult blood test; FIT, fecal immunochemical test.

**Table 1.** Characteristics of Study Cohort: Individuals With Negative Screening Colonoscopy After Positive FOBT Result

Characteristic	Individuals, No. (%) (N = 11 473)	
	Negative colonoscopy result (n = 8433)	Positive colonoscopy result (n = 3040)^a^
Sex		
Male	4288 (50.8)	1808 (59.5)
Female	4145 (49.2)	1232 (40.5)
Age, median (IQR), y	65 (63-67)	65 (63-67)
gFOBT	6400 (75.9)	1706 (56.1)
FIT	2033 (24.1)	1334 (43.9)
Quality indicators		
Adequate bowel preparation^b^	7946 (94.2)	2820 (92.8)
Missing, No.	23	5
Cecal intubation	8124 (96.3)	2873 (94.5)
Missing, No.	25	3
Photo of cecum	7739 (91.8)	2774 (91.3)
Missing, No.	1	0
Withdrawal time >10 min	8354 (99.1)	2993 (98.5)
Missing, No.	0	0

Abbreviations: gFOBT, guaiac fecal occult blood test; FIT, fecal immunochemical test.

^a^ Individuals with positive findings at screening colonoscopy for comparison.

^b^ Bowel preparation assessed by the endoscopist.

Among 8433 participants with negative colonoscopy results, 56 CRC cases were identified. Four cases were diagnosed within 6 months after the positive FOBT result; these were classified as screen-detected CRCs or identified during a parallel clinical investigation and were therefore excluded. During follow-up, 52 CRC cases occurred, including 29 cases (55.8%) in women and 23 cases (44.2%) in men. The total follow-up time was 62 619 person-years in the negative colonoscopy group. The median (range) time to CRC diagnosis was 4.8 (0.7-10.2) years. The median (IQR) time to diagnosis differed between men (4.1 [2.1-7.0] years) and women (5.7 [3.5-7.1] years) (Table 2).

**Table 2.** Characteristics of Individuals With CRC

CRC data	Individuals, No. (%)					
	Negative colonoscopy result			Nonadherence to follow-up colonoscopy		
	Total (n = 52)	Men (n = 23)	Women (n = 29)	Total (n = 56)	Men (n = 33)	Women (n = 23)
Type of FOBT^a^						
FIT	14 (26.9)	6 (26.1)	8 (27.6)	19 (33.9)	11 (33.3)	8 (34.8)
Median (IQR) FIT value	129 (91-678)	568 (177-3861)	98 (70-190)	158 (87-419)	281 (128-1103)	91 (60-118)
Quality indicators at index colonoscopy^b^						
Adequate bowel preparation	49 (94.2)	22 (95.7)	27 (93.1)	NA	NA	NA
Cecal intubation	50 (96.2)	23 (100)	27 (93.1)	NA	NA	NA
Photo of cecum	43 (82.7)	19 (82.6)	24 (82.8)	NA	NA	NA
Withdrawal time >10 min	51 (98.1)	22 (95.7)	29 (100)	NA	NA	NA
Screening detected	11 (21.2)	6 (26.1)	5 (17.2)	NA	NA	NA
Time to CRC diagnosis, y^c^						
Median (IQR)	4.8 (2.2-7.1)	4.1 (2.1-7.0)	5.7 (3.5-7.1)	2.8 (1.4-5.0)	2.2 (1.3-5.0)	3.4 (2.0-5.0)
0.5 to 2.0	8 (15.4)	4 (17.4)	4 (13.8)	22 (39.3)	16 (48.5)	6 (26.1)
>2.0 to 4.0	12 (23.1)	7 (30.4)	5 (17.2)	14 (25.0)	7 (21.2)	7 (30.4)
>4.0 to 6.0	11 (21.2)	5 (21.7)	6 (20.7)	8 (14.3)	2 (6.1)	6 (26.1)
>6.0 to 8.0	13 (25.0)	3 (13.0)	10 (34.5)	5 (8.9)	2 (6.1)	3 (13.0)
>8.0	8 (15.4)	4 (17.4)	4 (13.8)	7 (12.5)	6 (18.2)	1 (4.3)
Age at CRC diagnosis, y						
Median (IQR)	70 (67-73)	68 (66-72)	71 (68-74)	69 (67-71)	69 (66-70)	68 (67-71)
60-69	24 (46.2)	12 (52.2)	12 (41.4)	36 (64.3)	21 (63.6)	15 (65.2)
70-79	28 (53.8)	11 (47.8)	17 (58.6)	20 (35.7)	12 (36.4)	8 (34.8)
Tumor location						
Cecum and ascending colon	16 (30.8)	6 (26.1)	10 (34.5)	11 (19.6)	7 (21.1)	4 (17.4)
Transverse colon and flexures	6 (11.5)	2 (8.7)	4 (13.8)	8 (14.3)	4 (12.1)	4 (17.4)
Descending colon	3 (5.8)	1 (4.3)	2 (6.9)	1 (1.8)	0	1 (4.3)
Sigmoid colon	9 (17.3)	6 (26.1)	3 (10.3)	15 (26.8)	10 (30.3)	5 (21.7)
Appendix	2 (3.8)	1 (4.3)	1 (3.4)	1 (1.8)	0	1 (4.3)
Unspecified part of colon	4 (7.7)	0	4 (13.8)	0	0	0
Rectosigmoidal junction and rectum	12 (23.1)	7 (30.4)	5 (17.2)	13 (23.2)	7 (21.2)	6 (26.1)
Missing	0	0	0	7 (12.5)	5 (15.2)	2 (8.7)
TNM stage						
I	16 (30.8)	5 (21.7)	11 (37.9)	12 (21.4)	9 (27.3)	3 (13.0)
II	7 (13.5)	3 (13.0)	4 (13.8)	15 (26.8)	7 (21.2)	8 (34.8)
III	12 (23.1)	8 (34.8)	4 (13.8)	17 (30.4)	10 (30.3)	7 (30.4)
IV	14 (26.9)	5 (21.7)	9 (31.0)	7 (12.5)	4 (12.1)	3 (13.0)
Missing	3 (5.8)	2 (8.7)	1 (3.4)	5 (8.9)	3 (9.1)	2 (8.7)

Abbreviations: CRC, colorectal cancer; FIT, fecal immunochemical test; FOBT, fecal occult blood test; TNM, tumor, node, metastasis; NA, not applicable.

^a^ Refers to the first positive fecal occult blood test result before the negative follow-up colonoscopy result.

^b^ Defined as the first negative follow-up screening colonoscopy result.

^c^ Time to diagnosis started at 6 months from the mailing date of the fecal occult blood test result and continued until CRC diagnosis in the National Cancer Register.

The quality of the index colonoscopy was defined by bowel preparation, cecal intubation, withdrawal time, and photographic documentation of the cecum. Of 4 quality indicators, 3 were true in more than 94% of cases (eg, adequate bowel preparation: 49 of 52 cases [94.2%]), except for photographic documentation of the cecum, which was true in 43 cases (82.7%). CRC incidence remained decreased in sensitivity analysis restricted to high-quality procedures (SIR, 0.47; 95% CI, 0.34-0.64) (eTable 1 in Supplement 1).

During follow-up, 11 CRC cases were screen detected, diagnosed after reinvitation in the standard screening program after a positive FOBT result and a repeat colonoscopy. Among all 52 CRCs identified after a negative colonoscopy result, the most common stage was I (16 cases [30.8%]), followed by IV (14 cases [26.9%]). CRC incidence among participants with a negative colonoscopy result was lower than in the general population (SIR, 0.52; 95% CI, 0.39-0.68), with a lower SIR in men (0.37; 95% CI, 0.25-0.56) than women (0.71; 95% CI, 0.49-1.03) (Table 3). Results were similar compared with a population restricted to screening participants (SIR, 0.57; 95% CI, 0.43-0.75) (eTable 2 in Supplement 1). The cumulative hazard was 0.0096 for participants with a negative colonoscopy after 13.5 years.

**Table 3.** Standardized Incidence Rate of CRC Among Individuals With Positive FOBT Result

Group	CRC cases, No.	Person-years	Incidence rate, No. per 100 000 person-years	Expected CRC cases, No.	SIR (95% CI)	CRCs in population, No.^a^
Negative colonoscopy result						
Total	52	62 619	83	100.7	0.52 (0.39-0.68)	4423
Men	23	32 764	70	61.5	0.37 (0.25-0.56)	2431
Women	29	29 856	97	40.7	0.71 (0.49-1.03)	1992
Nonadherence to follow-up colonoscopy						
Total	56	8532	656	13.3	4.21 (3.24-5.48)	4423
Test type						
gFOBT	37	6751	548	10.8	3.43 (2.48-4.74)	4423
FIT	19	1781	1067	2.5	7.60 (4.84-11.94)	3434^b^
Sex						
Men	33	4751	695	8.6	3.84 (2.72–5.41)	2431
Women	23	3781	608	5.0	4.60 (3.05-6.94)	1988

Abbreviations: CRC, colorectal cancer; FIT, fecal immunochemical test; gFOBT, guaiac-based fecal occult blood test; SIR, standardized incidence ratio.

^a^ The general population was represented by all individuals in the cohort who were invited to screening, excluding the 2 groups described in this table.

^b^ The reference population differs between test types because of variations in age distributions given that FIT was introduced later during the study period.

In the group with a positive gFOBT or FIT result who did not adhere to follow-up screening colonoscopy, 100 CRC cases were diagnosed. However, 44 of these were diagnosed within 6 months of the mailing date of the positive FOBT result. All these cases were therefore excluded from the analysis because it is suspected that these individuals had initiated a clinical investigation before or during the screening process. In total, 56 CRC cases were then diagnosed among 1662 individuals during follow-up. The total follow-up time was 8532 person-years among those who did not adhere to follow-up screening colonoscopy. The median (range) time to diagnosis was 2.8 (0.5-12.2) years. The median (IQR) time was 2.2 (1.3-5.0) years for men and 3.4 (2.0-5.0) years for women. Most cancers were stage III (17 cases [30.4%]), and the most common location was sigmoid colon (15 cases [26.8%]). CRC incidence was significantly increased among individuals with a positive screening test who did not undergo the colonoscopy compared with the general population (SIR, 4.21; 95% CI, 3.24-5.48). The SIR was 3.84 (95% CI, 2.72-5.41) for men and 4.60 (95% CI, 3.05-6.94) for women. Results were similar compared with a population restricted to screening participants (SIR, 4.67; 95% CI, 3.58-6.08) (eTable 2 in Supplement 1). Stratified by test type, SIRs were higher among individuals screened with FIT (SIR, 7.60; 95% CI, 4.84-11.94) than those screened with gFOBT (SIR, 3.43; 95% CI, 2.48-4.74). The cumulative hazard was 0.0812 for individuals who did not adhere to follow-up colonoscopy after 13.5 years.

Among 8433 individuals with negative colonoscopy result, the median (IQR) FIT value was 118 (71-283) μg Hb/g of feces compared with 169 (94-430) μg Hb/g of feces among 3040 individuals with a positive colonoscopy result, which was a significant difference (*P* <.001) (Table 4). The difference in FIT value remained significant when separating men and women. Among participants with subsequent CRC, median (IQR) FIT values did not differ significantly between individuals with a prior negative colonoscopy result and those who did not adhere to follow-up colonoscopy (129 [91-678] vs 158 [87-419] μg Hb/g of feces; *P* = .70) (Table 2).

**Table 4.** FIT Values by Colonoscopy Result

Group	FIT result, median (IQR), μg Hb/g of feces		*P* value^a^
	Negative colonoscopy result	Positive colonoscopy result	
Total	118 (71-283)	169 (94-430)	<.001
Men	182 (108-438)	234 (128-515)	<.001
Women	83 (55-178)	120 (62-333)	<.001

^a^ Median FIT value was compared between groups using the Wilcoxon rank-sum test.

## Discussion

In this register-based prospective cohort study, individuals with a negative colonoscopy result had decreased CRC incidence compared with the general population (SIR, 0.52). The decrease in incidence was greater in men than in women. Given that no active intervention was performed with a negative colonoscopy, findings suggest that the procedure itself was not associated with a decrease in risk but rather identified a group with a lower baseline risk. In contrast, individuals with a positive FOBT result who did not undergo the follow-up colonoscopy had an increased incidence of CRC compared with the general population (SIR, 4.21).

Earlier findings have reported varying results regarding the risk of CRC after a negative screening colonoscopy result. These findings align with a 2025 US prospective cohort study^14^ based on self-reported data from the Nurses’ Health Study, which observed a 50% decrease in CRC incidence comparing a screened population with the general population. A similar decrease was observed in another retrospective cohort study from the US,^15^ although both studies included younger cohorts and used colonoscopy as the primary screening method. However, a Polish observational study^16^ involving opportunistic single-screening colonoscopies, potentially representing a healthier population, showed a less pronounced risk reduction. The quality of colonoscopies in our study was consistent with the definition of high-quality colonoscopy described in the Polish study.^16^ A Danish population-based cohort study^17^ examined CRC incidence after a negative colonoscopy result comparing individuals with positive vs negative FOBT results. The study found that a negative colonoscopy result after a positive gFOBT result within a screening program was associated with increased CRC incidence compared with negative results from colonoscopies performed in a clinical setting (adjusted hazard ratio, 1.86). Although colonoscopy quality was not reported in the Danish study, these findings underscore the challenges of comparing negative colonoscopy results across different clinical settings.

In this study, the observed incidence of CRC after a negative colonoscopy result compared with the general population was decreased, with a lower SIR in men than in women (0.37 vs 0.71). These findings are consistent with a Canadian retrospective, population-based cohort study.^18^ However, the Canadian study differed in design given that it involved a younger population, used colonoscopy as the primary screening modality, and excluded individuals with prior exposure to FOBT. A case-control study from Germany^19^ based on personal interviews and medical records also observed a more pronounced difference in CRC incidence among men. Moreover, a 2025 Danish study,^20^ which included screening colonoscopies with negative results in individuals with positive FIT results, highlighted concerns about current Danish screening guidelines recommending an 8-year reinvitation interval. Notably, the study found no statistically significant difference in CRC incidence among women after a negative colonoscopy result. A Swedish register-based study reported an increased incidence of CRC after a negative colonoscopy result among women compared with men in settings outside organized screening programs.^21^ The observed sex-based difference remains unexplained. One possible explanation is that advanced neoplasia has been observed more frequently in men than in women, which could be associated with a greater observed risk reduction in men after a negative colonoscopy result, potentially reflecting differences in baseline risk.^22^

Among individuals with positive screening tests in our study, 87.3% underwent colonoscopy, a rate considered high according to European guidelines.^23^ However, 12.7% did not proceed to colonoscopy, and this group demonstrated a markedly elevated SIR of 4.21 for CRC diagnosis during follow-up compared with the general population. These findings underscore the importance of improving participation rates in colonoscopy after a positive FOBT result. Targeted interventions to enhance follow-up adherence should be prioritized to effectively reduce CRC incidence.

FIT values were significantly elevated in individuals with a positive colonoscopy result compared with those whose colonoscopy result was negative. Additionally, among individuals with a negative colonoscopy result, men exhibited higher FIT values than women. These sex-based differences in FIT values, as well as the association between FIT values and colonoscopy outcomes, are consistent with findings from previous studies.^24,25^

This study has several strengths, including detailed data on colonoscopy quality and CRC diagnosis derived from nationwide registers with high coverage rates, thereby minimizing the risk of measurement errors. The study is also among few conducted within a strictly population-based FOBT screening program.

### Limitations

This study also has some limitations that should be acknowledged. Compared with previously cited studies, this study is smaller in scale. However, within the context of a population-based FOBT screening program, the sample size remains adequate, even though the outcome is rare and few events occurred. The cohort included individuals screened with both gFOBT and FIT, which may affect generalizability because FIT is now the standard method. In the negative colonoscopy group, the focus was on colonoscopy outcomes rather than prior test type. Among individuals who did not adhere to follow-up colonoscopy, we stratified CRC incidence by test type and observed an increased incidence in the FIT group, although the number of events was limited. The SIR calculation was adjusted for age, but we lacked information on other potential confounders that may influence cancer incidence. However, this was not the primary focus of our study given that the objective was to evaluate cancer incidence in the selected groups regardless of their baseline risk prior to participation in screening. A long follow-up period is essential when evaluating cancer incidence, and in this study, the longest possible follow-up was almost 14 years, encompassing a total of 71 151 person-years in the 2 cohorts. While an even longer follow-up would be ideal for assessing CRC incidence, this represents the longest feasible follow-up period given that CRC screening was introduced in the early 2000s.

## Conclusions

In this cohort study of CRC screening, we found that the incidence of CRC was significantly lower after a negative screening colonoscopy result, whereas individuals who did not adhere to colonoscopy after a positive FOBT result exhibited an increased incidence. Observed differences in incidence suggest that a risk-based, individualized approach to follow-up may be beneficial. In addition to colonoscopy findings, factors such as FIT levels, age, and sex could be incorporated into models for optimizing screening intervals. Given limited colonoscopy resources and the expansion of screening programs to include broader and younger populations, it is essential for screening efforts to be efficient. Resources should be allocated where they will have the greatest impact, and targeted strategies should be developed to engage high-risk individuals who do not attend follow-up procedures.

## Notes

Supplement 1. eTable 1. Sensitivity analysis with standardized incidence rate of CRC among individuals with positive FOBT result with a negative follow-up screening colonoscopy result restricted to colonoscopies with completed quality indicators eTable 2. Risk of CRC among individuals with positive FOBT result assessed in those with a negative follow-up screening colonoscopy result and those who did not adhere to follow-up screening colonoscopy

Supplement 2.
Data Sharing Statement
