# Supplementary material for: Colorectal Cancer Risk With Negative Colonoscopy or Nonadherence After Positive FOBT Screening
Source: JAMA Netw Open. 2026 Mar 19;9(3):e262404. doi: 10.1001/jamanetworkopen.2026.2404 (PMC13003367; doi:10.1001/jamanetworkopen.2026.2404)
Supplement: Supplement 1. — eTable 1. Sensitivity analysis with standardized incidence rate of CRC among individuals with positive FOBT result with a negative follow-up screening colonoscopy result restricted to colonoscopies with completed quality indicators eTable 2. Risk of CRC among individuals with positive FOBT result assessed in those with a negative follow-up screening colonoscopy result and those who did not adhere to follow-up screening colonoscopy [file jamanetwopen-e262404-s001.pdf]

Supplemental Online Content

Heyman H, Saraste D, Jonsson H, Blom J. Colorectal cancer risk with negative colonoscopy or nonadherence after positive FOBT screening. *JAMA Netw Open*. 2026;9(3):e262404. doi:10.1001/jamanetworkopen.2026.2404

eTable 1. Sensitivity analysis with standardized incidence rate of CRC among individuals with positive FOBT result with a negative follow-up screening colonoscopy result restricted to colonoscopies with completed quality indicators

eTable 2. Risk of CRC among individuals with positive FOBT result assessed in those with a negative follow-up screening colonoscopy result and those who did not adhere to follow-up screening colonoscopy

This supplemental material has been provided by the authors to give readers additional information about their work.

© 2026 Heyman H et al. *JAMA Network Open*.

eTable 1. Sensitivity analysis with standardized incidence rate of CRC among FOBT positive individuals with a negative follow-up screening colonoscopy restricted to colonoscopies with completed quality indicators (n=7,301).

	Number of CRC	Person-years	Incidence rate (per 100,000 PY)	Expected CRC cases	SIR (95% CI)	Number of CRC in population
Negative colonoscopy	40	53,407	75	85	0.47 (0.34-0.64)	4,423

eTable 2. Risk of CRC among FOBT positive individuals was assessed in two groups: those with a negative follow-up screening colonoscopy and those who did not adhere to follow-up screening colonoscopy.

	Number of CRC	Person-years	Incidence rate (per 100,000 PY)	Expected CRC cases	SIR (95% CI)	Number of CRC in population[a]
Negative colonoscopy						
Total	52	62,619	84	91.6	0.57 (0.43-0.75)	2,793
Men	23	32,764	70	54.4	0.42 (0.28- 0.64)	1,401
Women	29	29,856	97	38.8	0.75 (0.52-1.08)	1,392
Non-adherence to follow-up colonoscopy						
Total	56	8,532	656	12	4.67 (3.58- 6.08)	2,793
Men	33	4,751	695	7.6	4.34 (3.07-6.13)	1,401
Women	23	3,781	608	4.7	4.89 (3.24-7.39)	1,389

Abbreviations: CRC, colorectal cancer; SIR, standardized incidence ratio; CI, confidence interval.
[a] The general population was represented by all individuals in the cohort who participated in screening, excluding the two groups above.

© 2026 Heyman H et al. *JAMA Network Open*.
