# Supplementary material for: Colorectal Cancer Risk With Negative Colonoscopy or Nonadherence After Positive FOBT Screening
Source: JAMA Netw Open. 2026 Mar 19;9(3):e262404. doi: 10.1001/jamanetworkopen.2026.2404 (PMC13003367; doi:10.1001/jamanetworkopen.2026.2404)
Supplement: Supplement 2. — Data Sharing Statement [file jamanetwopen-e262404-s002.pdf]

Data Sharing Statement

Heyman. Colorectal Cancer Risk With Negative Colonoscopy or Nonadherence After Positive FOBT Screening. *JAMA Netw Open*. Published online March 19, 2026. doi:10.1001/jamanetworkopen.2026.2404

Data
Data available: No
